# Computational Simulation of Respiration-Induced Deformation of Renal Arteries After EVAR

**DOI:** 10.1007/s10439-025-03806-y

**Published:** 2025-07-22

**Authors:** Alessandra Corvo, Stéphane Avril, Alberto Aliseda, Stéphan Haulon, Fanette Chassagne

**Affiliations:** 1 Mines Saint-Etienne, INSERM, U1059 SAINBIOSE, F42023 Saint-Etienne, France; 2 Department of Mechanical Engineering, University of Washington, Seattle, WA 98105 USA; 3 Aortic Center, Marie Lannelongue Hospital, Groupe Hospitalier Paris Saint Joseph, Paris Saclay University, 92350 Le Plessis-Robinson, France

**Keywords:** Abdominal aortic aneurysm, EVAR, Stent-graft, Breathing, Numerical simulations

## Abstract

****Purpose**:**

Fenestrated endovascular aneurysm repair (fEVAR) is widely used to treat complex abdominal aortic aneurysms, requiring renal artery stenting. However, complications such as occlusion can occur within the renal arteries. This study examines the effect of respiration-induced deformations, using patient-specific models and computational simulations. By investigating the impact of stenting and breathing, this research aims to improve surgical pre-planning and minimize EVAR complications.

****Methods**:**

Pre-EVAR geometries from CT scans were segmented and meshed. Respiratory-induced displacements were applied to the segmented ends of the renal arteries to simulate breathing. The deployment process was achieved via balloon expansion, testing bridging stent-grafts with different lengths. To evaluate the accuracy of the workflow, simulated results and post-op CT scans were compared using centerline analysis, measuring morphological differences between the patient-specific models and the actual patients.

****Results**:**

Numerical simulations accurately predicted renal artery movement during respiration, aligning with in vivo measurements. Simulated stent-graft configurations closely matched post-EVAR CT scans. Stent-graft protrusions into the aortic lumen were within the expected range, indicating correct positioning. Longer stent-grafts constrained renal artery movement, affecting branching angle changes, while shorter grafts had a less pronounced impact.

****Conclusions**:**

Our novel digital twin model accurately simulates fEVAR procedures, including the deployment of renal bridging stent-grafts. Numerical simulations capture the bending of the renal arteries during breathing and their morphological changes following stenting in the post-operative configurations. Future research aims to expand the patient cohort and combine the solid mechanics simulations with CFD analysis.

## Introduction

Abdominal Aortic Aneurysms (AAA) are dilatations of the abdominal aorta of more than 3 cm in diameter or more than 50% of its diameter. This pathology is the consequence of changes in the biological composition of the arterial wall, such as the fragmentation of elastin in the intima [1, 2] and is associated with many risk factors such as male gender, aging, vascular diseases, hypertension, smoking, family history, hypercholesterolemia [3], or connective tissue disorders [1]. AAA rupture, resulting in internal hemorrhage, accounts for 5 deaths/100,000 and 2 deaths/100,000 in men and women, respectively, in USA and Europe, for a total of around 167,000 deaths and 3 million disability-adjusted life years worldwide [4, 5]. In clinical practice, when the maximum aneurysm diameter exceeds the limit of 5 cm, patients undergo surgical open repair or endovascular aneurysm repair (EVAR) to prevent rupture [1]. This latter surgical treatment is a minimally invasive intervention based on stent-graft (SG) deployment inside the abdominal aorta to prevent blood flow from entering the AAA, minimizing surgical trauma and recovery time for the patients. When the AAA involves other arterial branches, fenestrated stent-grafts are deployed, including holes (fenestrations) to allow blood flow through stent-graft wall and into the branched arteries [6]. Since the exact position of fenestrations is specific for each single patient, fenestrated aortic stent-grafts are custom-made and based on patient imaging. After aortic stent-graft placement, peripheral stent-grafts are deployed through the fenestrations in the peripheral branches, such as the renal arteries, to restore normal blood flow through these branches. Despite the advantages of this surgical procedure, kinking and occlusions occur for 2–4% of patients following EVAR [7]. More specifically, if the fEVAR involves the renal arteries, occlusions leading to acute renal failure were reported in 6.7% of patients [8] and this incidence could be under-reported [9].

The prediction of the outcome of the surgical repair would be extremely useful, potentially avoiding life-threatening complications for the patient. For this, pre-treatment numerical simulations can support the surgeon’s stent choice and define the stent length [10]. For fenestrated EVAR, the most complex clinical choice concerns the SG length and diameter: endograft insufficient oversizing is indeed associated with stent migration and higher risk of endoleaks [11, 12]. Conversely, excessive oversizing may induce crimping of the graft material within the lumen, potentially causing occlusions, or rupture of the vessel [13]. Specifically for bridging stent-grafts, adverse events were related to an insufficient landing zone or to imprecise planning of the procedure [14]. Computational biomechanics can contribute to the optimization of SG design and EVAR outcomes [15].

Only a few cases in the literature report simulations on patient-specific geometries [16–19]. Auricchio et al. published the first study with EVAR simulation on a patient-specific model of ascending aortic aneurysm [15]. De Bock et al., Prasad et al., Jayendiran et al., Perrin et al., Hemmler et al., and Dupont et al., Sanford et al. performed numerical simulations of stent-graft deployment in AAAs [16–22], but only the last four performed complete EVAR simulations on patient-specific geometries [16–19].

Respiration is a factor that may contribute to EVAR complications. During breathing, the diaphragm contraction and relaxation induce motion of abdominal organs, especially in the cranio-caudal direction. This implies translation, bending or axial deformation of the visceral arteries, such as the left (LRA) and right renal arteries (RRA). Suh et al. investigated breathing-induced motion and deformation of the renal arteries [23]. They reported values of change in mean and maximal curvature as well as changes in branching angle (defined as the angle defined by the centerlines of the aorta and the branching artery) of the renal arteries for AAA patients, caused by respiratory motion. Tran et al. and Cheng et al. were the first to evaluate the respiration-induced changes in branch vessels for patients undergoing EVAR, with fenestrated and snorkel stent-grafts, respectively, by comparing pre- and post-operative images [24, 25].

Bending of the renal arteries could affect renal stenting and induce complications like restenosis and renal occlusions. However, despite renal occlusions severely compromising kidney function and thus being life-threatening, the mechanism leading to this complication is not fully understood, making it difficult to predict. To the authors’ knowledge, no studies have numerically simulated renal artery movement due to breathing and its impact on SG deployment. The aim of this study is to use patient-specific simulation to understand the effect of breathing on renal artery deformation and its subsequent effect on stent-grafts deployment. This patient-specific model is validated against post-operative imaging. The breathing simulation is conducted for three different lengths of stent-grafts, considering the nominal length, a shorter option, and a longer one. The goal is to assess how the morphology of the renal arteries change when a different stent-graft is introduced, including respiratory motion. Ultimately, this could provide endovascular surgeons with recommendations on the outcomes of different commercial stent-grafts implantation that minimize the risk of complications.

## Materials and Methods

### Modeling of the Aorta and Renal Arteries

#### Geometries of the Aorta and Renal Arteries

Two patients who underwent EVAR at Marie Lannelongue Hospital (Paris) were included in this study (enrolled patients signed a dedicated informed consent); CT scans were collected pre- and post-operatively (on average, 31 days after the intervention) and at two times during the respiratory cycle, in breath-hold conditions: inspiration and expiration. Via a semi-automatic segmentation, geometrical models including the abdominal aorta and the renal arteries were created from the pre-operative imaging. Minimal smoothing was applied to the segmented geometries (0.5 as smoothing factor).

#### Mechanical Modeling of the Aorta and Renal Arteries

Following segmentation, to ensure a good mesh quality for patient-specific geometries, the geometries were meshed with triangular shell elements [16, 18, 19, 21], with the same element size as previously validated studies (1.5 mm mean edge length) [16], for a total of approximately 48,000 elements. A wall thickness of 1.5 mm was specified for the aortic walls [16] and 0.5 mm for the renal arteries [26, 27]. The arterial wall was modeled with an anisotropic Holzapfel–Gasser–Ogden model as in previous studies [16, 28].

### Modeling of the Stent-Graft

#### Stent-Graft Geometry

**Fig. 1:**
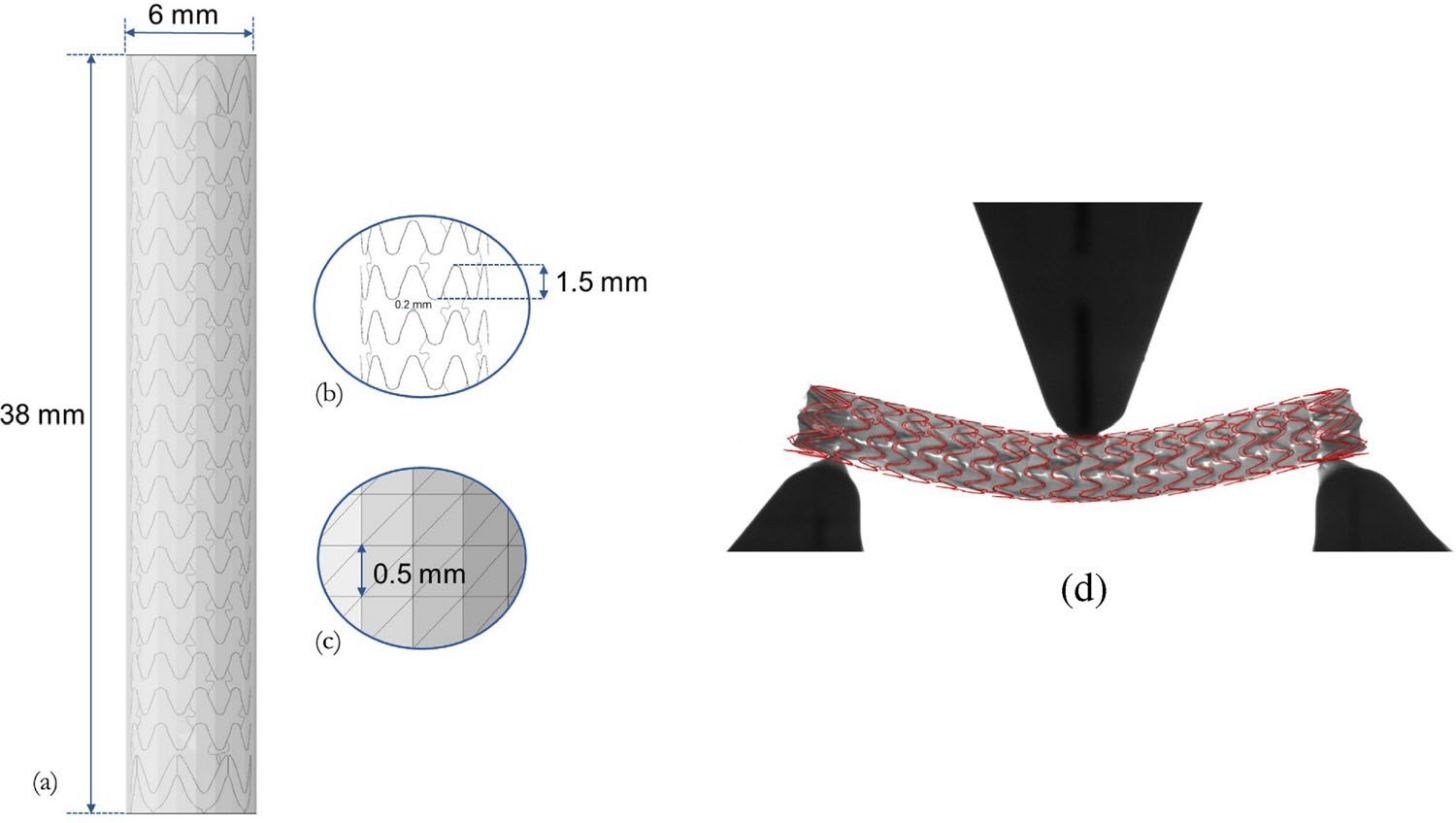
**a** Peripheral SG model (6 $\times$ 38 mm) with transparency applied to show the internal stent details; **b** zoomed view of the stent details, including strut height and beam element length; **c** detail of the graft mesh with indication of the element size. **d** 3-Point bending: visual comparison between the in silico (red) and experimental configurations (background). The graft has been removed to facilitate the visualization and stent positioning comparison

Two commercial peripheral SG samples were characterized. $\upmu$-CT scans were performed on a stent-graft, to obtain its three-dimensional geometry. From this, an STL model of a peripheral SG was generated through the ‘Wrap mesh’ tool available in Abaqus (Dassault Systèmes Simulia Corp., Providence, RI, USA). This geometry was then modified to generate the different stents according to the length and diameter that are commercially available (Fig. 1a–c).

#### Mechanical Characterization of the Stent-Graft

The stent was modeled using B31 beam elements (0.2 mm mean length) with a 0.115 mm by 0.145 mm rectangular profile, as determined from the segmentation of the $\upmu$-CTs. The stent is made of cobalt–chromium (Co–Cr) alloy exhibiting an elasto-plastic behavior, with a Young Modulus of 268 GPa, and a Poisson ratio equal to 0.3 in the elastic phase. To simulate the balloon expansion of the stent-graft, the peripheral stent-graft was modeled as an elasto-plastic material, based on the tensile stress–strain curve provided for Co–Cr L605 alloy [29]. The polymeric part (graft) is composed of expanded polytetrafluoroethylene (ePTFE). As the mechanical properties of these stent-grafts, particularly the graft component, are not well documented in the literature, mechanical characterization of two samples of graft raw material was performed. The graft was modeled with S3 triangular shell elements (0.2 mm shell thickness) with a mesh element size of 0.5 mm. The stent and the graft were automatically meshed on Abaqus, imposing the aforementioned mesh sizes. The graft was positioned on the stent by aligning their centroids, and, by applying a radial displacement, the graft was placed onto the stent, taking into account their different thicknesses. By implementing a tie constraint on Abaqus, all of the nodes of the stent are tied to the graft.

The lamina option was selected to account for the orthotropic behavior of the graft, defined by six parameters: $E_1$, $E_2$, $\nu _{12}$, $G_{12}$, $G_{13}$, and $G_{23}$. To identify these mechanical parameters, starting with the Young’s Modulus $E_1$ and the Poisson ratio, a uniaxial tensile test was conducted on a sample of the raw graft material using an Instron 3343 machine (Norwood, MA, USA) at room temperature, coupled to a Digital Image Correlation (DIC) system including two high-speed cameras (Photron UX100 mini with 105 mm macro lenses). A speckle pattern was sprayed using black paint. The sample (38 mm × 6 mm) was secured in the pneumatic grips of the Instron and stretched for five cycles up to a displacement of 3 mm, at a velocity of 0.1 mm/s. The cameras of the DIC system captured the sample at a sampling rate of 50 Hz. Stress was computed as the reaction force divided by the initial cross-sectional area of the sample. Strains were derived from the grip’s displacement. Excluding data from the first load cycle and the initial values corresponding to the preload phase, the slope of the stress–strain curve was calculated to derive the Young modulus $E_1$ in the longitudinal direction, found to be equal to 1200 MPa (Table 1). The values of transverse strains were derived by using the VIC-3D software (Correlated solutions, Columbia, USA). By solving the constitutive equations, the value of the Poisson ratio was calculated in the region of interest. Based on these calculations, the Poisson ratio was determined to be 0.16 (Table 1).

**Table 1:** Stent-graft material properties

Stent	(CoCr)	Graft	(ePTFE)
E	268 GPa	$E_1$	1.2 GPa
		$E_2$	1.8 MPa
$\nu$	0.3	$\nu _{12}$	0.16
		$G_{12}$	1 MPa
		$G_{13}$	60 MPa
		$G_{23}$	35 MPa

#### Validation Through 3-Point Bending Test

To define the remaining mechanical parameters required to model the graft’s mechanical properties, three-point bending tests were conducted on a SG measuring 38 mm in length and 6 mm in diameter at room temperature, following the protocol of the standard ASTM F2606-08 [30]. To prevent plastic deformation, the maximum deflection applied was limited to 3 mm, with a displacement rate of 0.1 mm/s for 5 cycles. The evolution of the configuration of the stent-graft during the test was recorded with a camera (Fig. 1d). A numerical model of the experiments was implemented in Abaqus (Fig. 1d). The three supports were modeled as rigid cylinders and a displacement of 3 mm was imposed on the reference point of the upper cylinder. The general contact algorithm was used, with a friction coefficient between the cylinders and the stent-graft set to 0.2. Different input parameters (except for $E_1$ and $\nu _{12}$) for the graft lamina model were tested to match the experimental data. The force/displacement curves obtained numerically and experimentally were compared. The identified material parameters $E_2$, $G_{12}$, $G_{13}$, and $G_{23}$ are presented in Table 1.

### Boundary Conditions for SG Deployment

The main body of the fenestrated aortic SG was customized to the patient’s anatomy, including fenestrations at the ostium of the renal arteries, the superior mesenteric artery (SMA), and the celiac artery. The self-expandable aortic stent-graft was then crimped, and deployed in the abdominal aorta (initially straightened using a morphing algorithm), following a validated workflow [16].

**Fig. 2:**
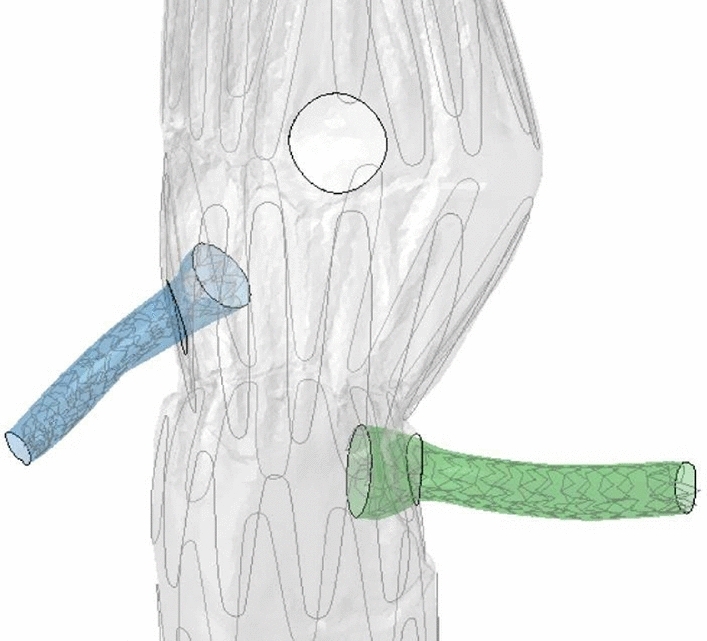
Renal stent-grafts after deployment for the right renal artery (blue) and the left renal artery (green). The peripheral stent-graft segment protruding into the main stent-graft body (gray) has been further expanded to match the fenestration diameter. The aorta and renal arteries have been removed from the plot for better visualization

Then, to deploy the balloon-expandable peripheral SG within the renal arteries, the device was crimped by imposing a radial displacement to all nodes of stent and graft, while keeping the other degrees of freedom fixed. To ensure the attachment of the stent to the graft, a tie constraint was applied between the two contact surfaces, and a contact interaction was defined with a penalty factor (f = 0.2). Once the stent-graft was correctly positioned, the renal artery was straightened to facilitate the deployment of the stent-graft. During the equilibrium phase, the crimped configuration of the peripheral stent-graft was imported from the crimping step and a balloon was placed inside the crimped SG, both aligned with the direction of the straightened renal artery and positioned inside the renal arteries. Based on previous studies, the balloon was modeled as a cylinder with linear elastic material properties (E = 900 MPa, $\nu$ = 0.3) and a thickness of 0.5 mm [31]. A radial dilation was imposed to the nodes of the balloon and contact was activated between the balloon and the stent with a friction coefficient of 0.2, according to similar studies [32]. The friction coefficient between the SG and the renal artery was 0.1 [33–35]. Thanks to the dilation of the balloon, the SG was radially expanded until its diameter matched that of the artery, approximately between 5 and 7 mm. The stent-graft was then correctly placed inside the artery, releasing all degrees of freedom, while keeping the renal artery end fixed. In the operating room, a second balloon may be introduced to inflate the extremity of the stent-graft, adjusting its diameter to the fenestration of the aortic SG main body. This step ensures a minimal oversizing for the bridging stent-graft protrusion into the aortic lumen and secures the stent-graft to prevent its migration. When required in the simulations, the balloon was further inflated by applying additional pressure exclusively to its inner surface at the level of the protrusion until its diameter matched the fenestration (Fig. 2). The artery was subsequently re-morphed to its original conformation, and an additional step was added to relax the stresses induced by the morphing, ensuring a new equilibrium between the bridging stent-graft and the artery.

### Boundary Conditions for the Respiratory Motion

The effect of breathing was numerically simulated in the pre-operative and post-operative geometry by applying a displacement in three directions (cranio-caudal, antero-posterior, and left–right) at the reference point (RP) of the distal end of the renal arteries (Fig. 5a). Proximal and distal ends of the aorta were fixed, assuming no motion of the aorta in those regions during breathing. The displacement vector was based on measurements from the patients’ CT scans from inspiration to expiration. Identical boundary conditions are applied after the deployment of the stent-graft, to assess the differences on artery deformation and stresses during respiration due to the presence of the device. The details of the imposed boundary conditions were added to the Appendix.

### Simulation Setup

To simulate the deployment of peripheral SGs in the renal arteries of the two patients, stent-grafts were initially designed to match the length and diameter of the devices implanted in the operating room (4 nominal SGs, left and right renal arteries for each of the 2 patients). To assess the impact of SG length on the artery displacements, and on potential complications, four additional SGs were tested for each patient (2 for the left, 2 for the right renal artery), corresponding to lengths both shorter and longer than the ones actually implanted. The details of the designed stent-grafts, including diameter and length, are provided in Table 2. All sizes correspond to commercially available stent-grafts.

**Table 2:** Bridging SG geometrical properties for Patient 1 and Patient 2

		Right renal artery		Left renal artery	
		Length (mm)	Diameter (mm)	Length (mm)	Diameter (mm)
	Nominal SG	23	7	22	5
Patient 1	Shorter SG	18	7	18	5
	Longer SG	27	7	28	5
	Nominal SG	22	6	22	5
Patient 2	Shorter SG	18	6	18	5
	Longer SG	28	6	28	5

**Fig. 3:**
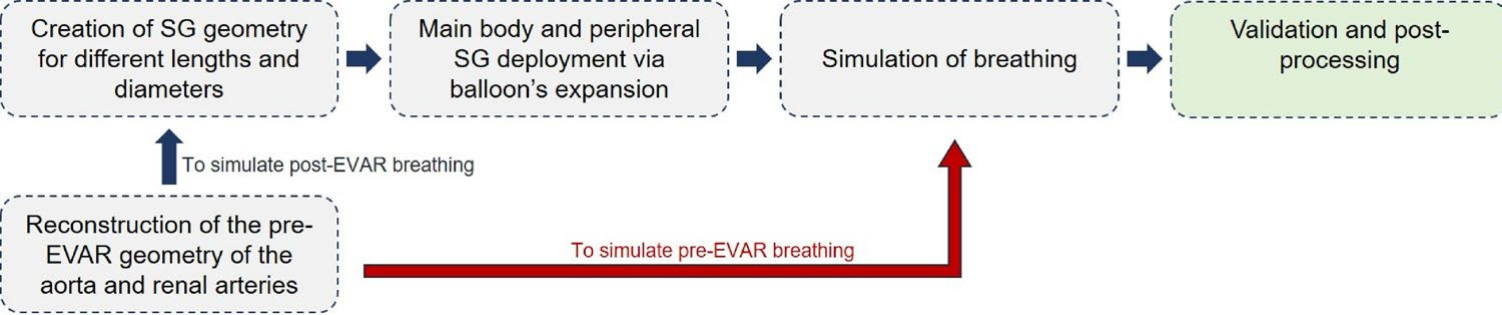
Simulation workflow to simulate pre-EVAR breathing (red) and post-EVAR breathing (blue)

The steps of the entire simulation workflow are summarized in Fig. 3.

### Validation and Post-processing

**Fig. 4:**
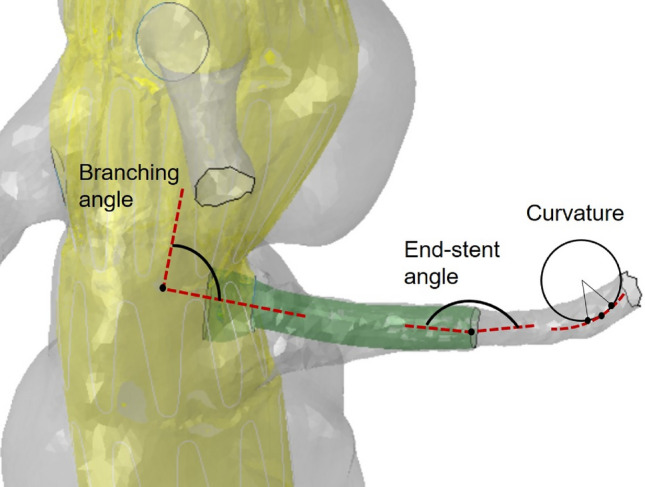
Detail of the patient-specific geometry with aortic main body and flared stent-graft. Morphologic metrics are shown: branching angle (angle between centerline of the renal artery and centerline of the aorta), end-stent angle (angle between the centerline of the stented and unstented artery), curvature (inverse of the radius of curvature)

To validate the simulations workflow, vessel centerlines were extracted using the Vascular Modeling ToolKit (VMTK). Specifically, variations in the branching angle and maximum curvature were computed using MATLAB. The branching angle is the angle measured between the centerline of the aorta and the centerline of the renal artery (Fig. 4); a perpendicular branch orientation corresponds to $90^\circ$ branching angle [24]. The curvature of the centerline is computed every 1 mm, and the maximum value along this line is considered in the analysis (Fig. 4) [24]. The end-stent angle is the angle measured between the centerline of the stented artery and the centerline of the unstented renal artery (Fig. 4). The difference in branching angle and end-stent angle from inspiration to expiration, as well as the difference in renal artery maximum curvature, were computed to provide a quantitative evaluation of morphological changes due to breathing. The collected data were then compared with in vivo results reported in the literature [23, 24] for pre-EVAR and post-EVAR configurations. The same procedure was applied to evaluate the effect of breathing for the simulations post-EVAR. In vivo data reported in the literature belong to datasets consistent with ours and therefore suitable for comparison, in terms of both age (average of 73 years) and gender (male patients).

To ensure that the predicted conformation of the stent-graft matched the actual outcome of the intervention, only the aortic stent and the two SGs at the renal level were segmented from post-operative CT scans, using the 3D Slicer software. The geometries of the aortic grafts and peripheral grafts resulting from the numerical simulations of SG deployment were then extracted. A qualitative comparison between STL models segmented from the CT scans and the FE simulations was performed to validate the simulation workflow. For the registration of the geometries, an Iterative Closest Point algorithm was implemented in Python, after an initial alignment of their centroids. To quantitatively assess the accuracy of the numerical simulations at predicting the post-EVAR geometry, the centerlines of the post-EVAR renal artery lumen and the centerlines of the graft resulting from the simulation were extracted. After interpolation, the distance between the corresponding points of the centerlines was measured. Additionally, the length of the peripheral stent-graft protrusion into the aorta after the complete deployment was compared to clinical recommendations.

## Results

### Validation of Respiration Simulation

**Fig. 5:**
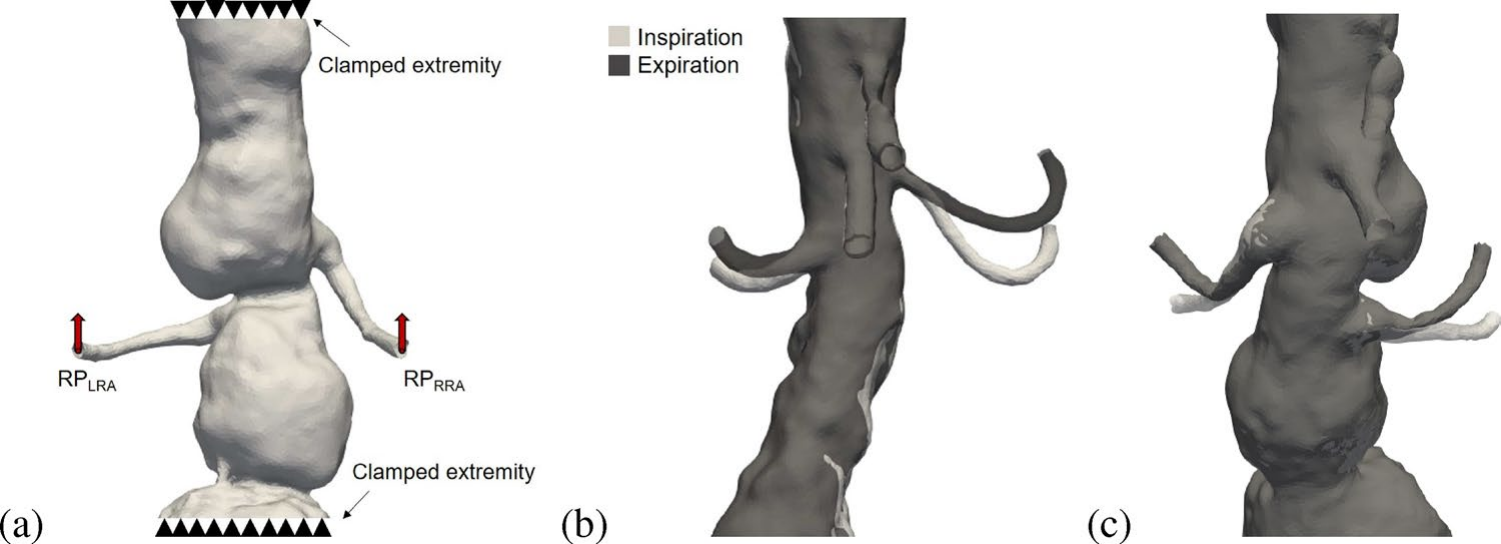
Boundary conditions for respiratory motion. Red arrows indicate the displacement applied mainly in the cranio-caudal direction for the reference point (RP) of the right and left renal artery (**a**). Proximal and distal ends of the aorta are fixed. Results of the simulation of breathing: inspiration configuration (light gray), expiration configuration (dark gray) for right renal artery (left) and left renal artery (right) for Patient 1 (**b**) and Patient 2 (**c**)

A visual representation of the results of the breathing simulation for Patients 1 and 2, in pre-operative configuration, is provided in Fig. 5. The light gray color indicates the configuration of the renal arteries during inspiration, while the dark gray represents the configuration during expiration, where the cranio-caudal movement of both renal arteries and their bending are clearly evident.

**Fig. 6:**
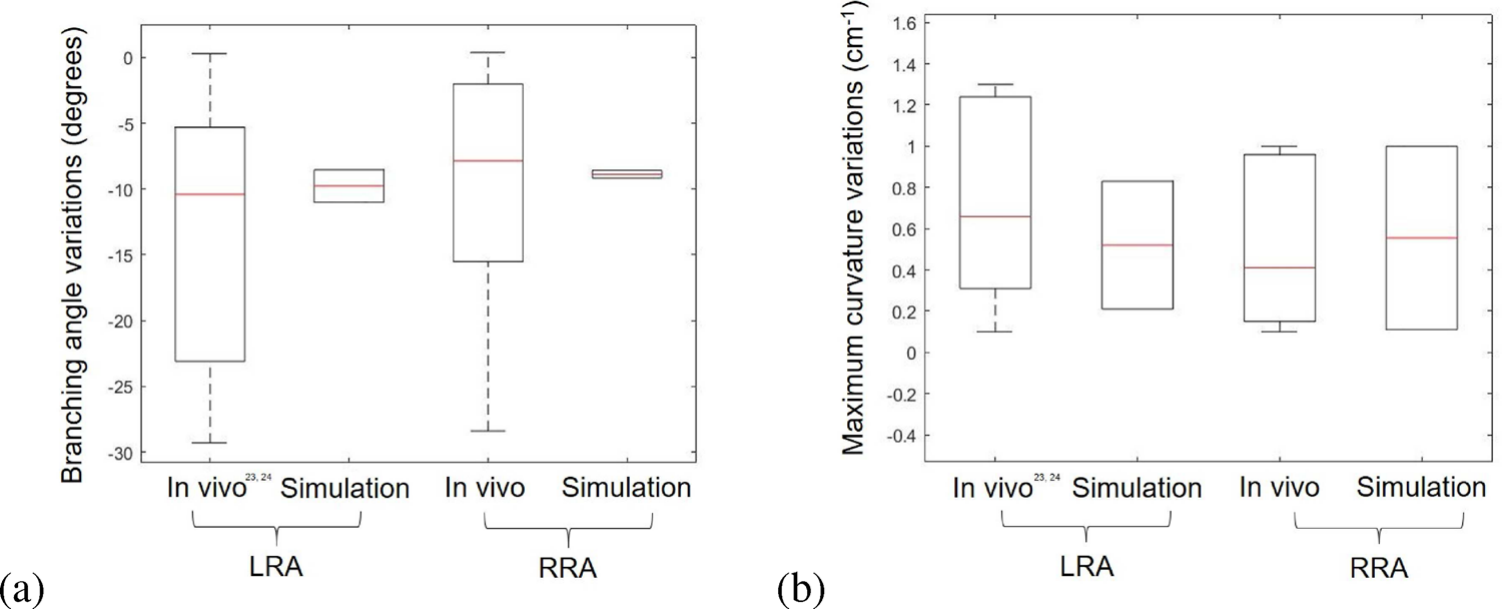
Branching angle (**a**) and maximum curvature (**b**) changes in renal artery due to breathing (from inspiration to expiration): comparison between in vivo measurements [23, 24]

The differences in branching angle and maximum curvature from inspiration to expiration for the renal arteries of the two patients in the pre-EVAR configuration are presented in Fig. 6. These values were compared with in vivo measurements from Suh et al. [23] and Tran et al. [24].

For both parameters, the data obtained from the two patients are within the range observed in previous studies, demonstrating the efficacy of the simulations in predicting the position and the bending of the renal arteries in inspiration and expiration for the two patients.

### Qualitative Comparison of Deployed Peripheral Stent-Graft

**Fig. 7:**
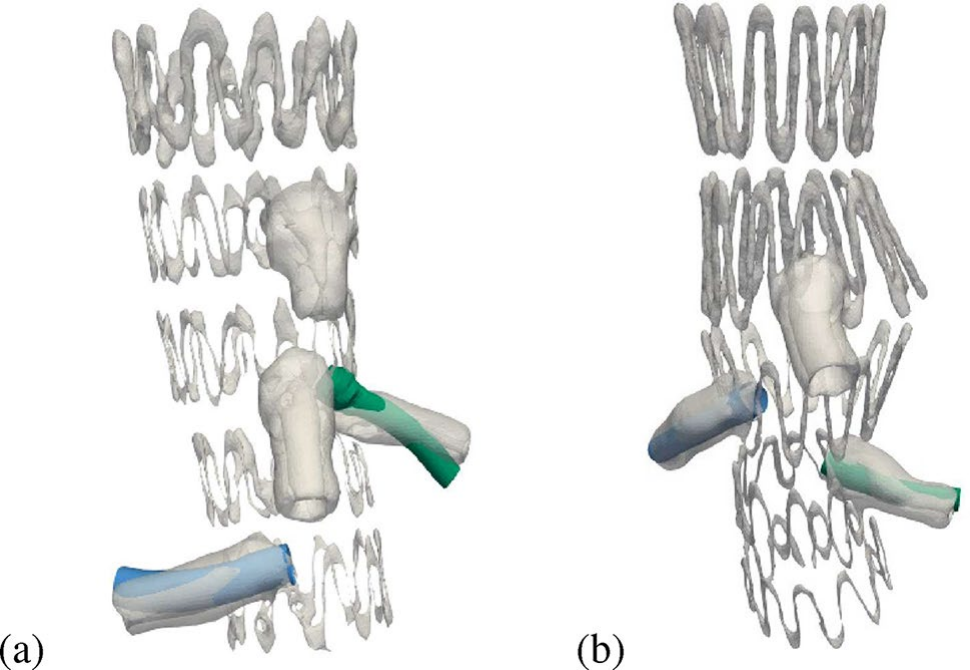
Qualitative comparison between the segmented aortic stent from post-operative imaging (gray) and peripheral grafts extracted from simulations (blue for RRA, green for LRA) for Patient 1 (**a**) and Patient 2 (**b**)

The SG models resulting from post-EVAR CT scans are presented in Fig. 7, along with the graft geometries extracted from the numerical simulations for Patient 1 and Patient 2. As shown in Fig. 7, a good agreement is observed between the two configurations of the stent-grafts. However, more noticeable differences can be seen in the curvature of the graft. Specifically, in the case of the right renal artery of Patient 1, the graft shape resulting from the simulations is slightly more curved than the model obtained from segmentation. Similar discrepancy can be observed for Patient 2 in Fig. 7b.

### Quantitative Comparison of Deployed Peripheral Stent-Graft

After extracting the centerlines of the renal arteries and the graft in its deployed configuration, the distance between centerline points was calculated. The average distance between the points, along with the standard deviation and the maximum distance, is presented in Table 3. A tolerance threshold of 3 mm was set to validate the efficacy of the simulations, taking into account 2 mm due to potential errors in image acquisition (misregistration, patient movement) [36], plus 1 mm related to segmentation errors, accounting for a margin of error equal to 1/3 of the mean radius (3 mm) of the renal arteries of the patients included in the study.

**Table 3:** Quantitative comparison of deployed peripheral stent-graft with nominal length for Patients 1 and 2 with respect to post-op imaging

Case	Artery	Distance between centerlines points	(mm)	SG Placement Accuracy Score (%)
		Mean	1.59	100
Patient 1	RRA	Std deviation	$\pm 0.48$	
		Max distance	2.43	
		Mean	2.90	45
Patient 1	LRA	Std deviation	$\pm 0.73$	
		Max distance	4.47	
		Mean	1.70	93
Patient 2	RRA	Std deviation	$\pm 0.71$	
		Max distance	3.70	
		Mean	2.34	80
Patient 2	LRA	Std deviation	$\pm 0.6$	
		Max distance	3.51	

A score to assess the effectiveness of simulations in predicting the exact placement of the stent-graft was defined (SG Placement Accuracy Score). This score corresponds to the percentage of points of the simulation-extracted centerline below the threshold of 3 mm out of the total of points of the centerline (Table 3).

### Validation of Peripheral SG Positioning into the Aortic Lumen

To verify the correct positioning of the peripheral stent-grafts in the renal arteries, the length of the bridging SGs protruding into the aortic lumen was measured (Table 4). A recent study conducted on patients treated with fEVAR indicated that the median length for the SG protrusion varied from 3.6 to 4.1 mm [37]. To validate the outcomes of our simulations, a marginally wider range was considered (3.4–4.3 mm) to account for a margin of error in the deployment simulation. All measured lengths fall within the aforementioned range (Table 4) and, particularly, the average length of the bridging SGs protruding into the aortic lumen was found to be 3.95 mm, aligning with the reference value of 3.88 mm identified as the standard in renal arteries for vascular surgeons [37].

**Table 4:** Length of bridging SG protrusion into the aortic lumen

	Length of SG protrusion into the aortic lumen for nominal length	(mm)
Patient 1	RRA	4.27
	LRA	4.01
Patient 2	RRA	3.90
	LRA	4.01

	Length of SG protrusion into the aortic lumen for shorter length	(mm)
Patient 1	RRA	3.51
	LRA	4.02
Patient 2	RRA	3.68
	LRA	3.63

	Length of SG protrusion into the aortic lumen for longer length	(mm)
Patient 1	RRA	4.27
	LRA	4.00
Patient 2	RRA	4.01
	LRA	4.12

### Effect of Breathing in Post-EVAR Configuration

**Fig. 8:**
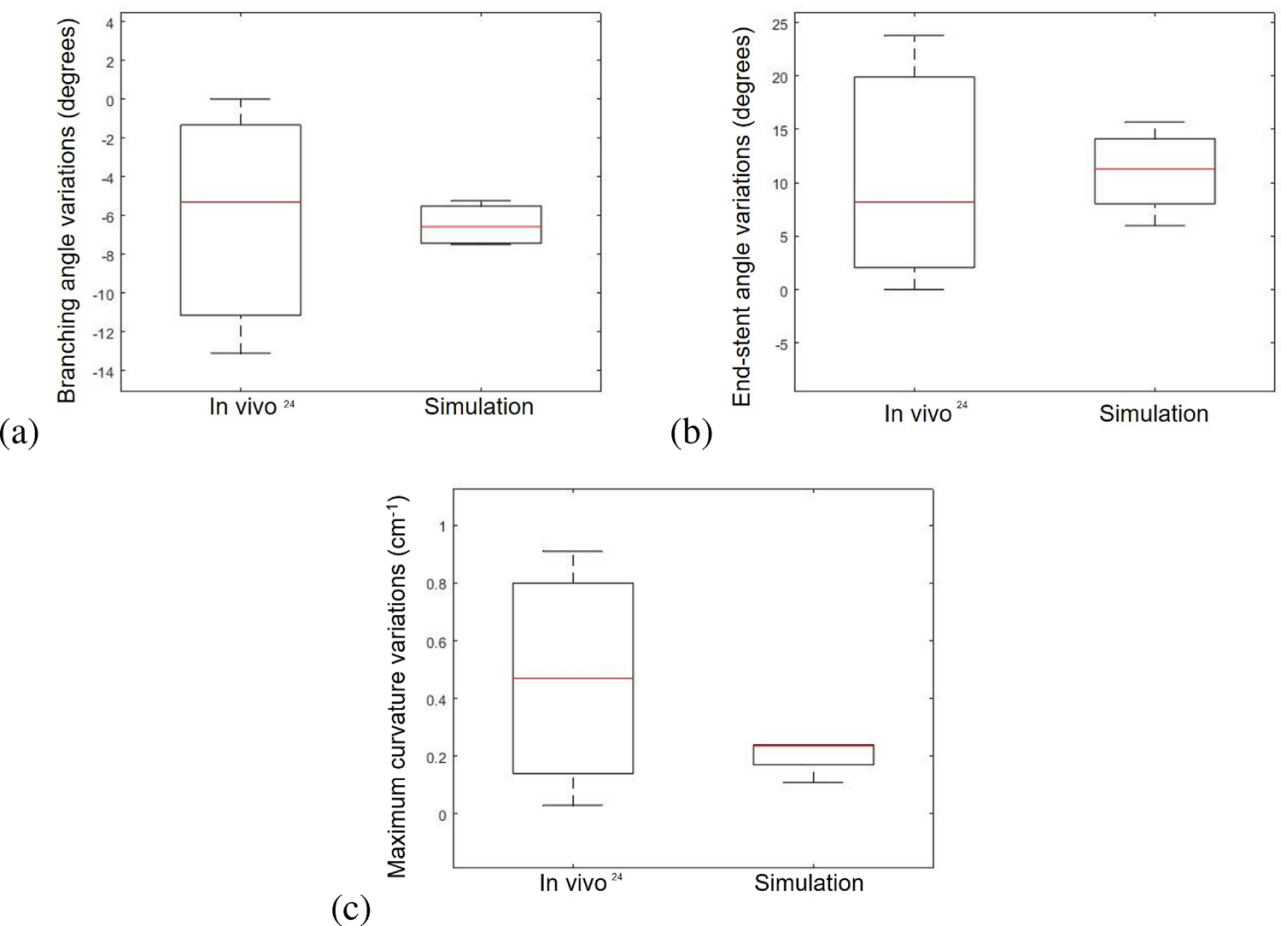
Branching angle (**a**), end-stent angle (**b**), and maximum curvature (**c**) changes in renal artery due to breathing: comparison between in vivo measurements [24] and in silico results in the post-EVAR configuration

The variations in geometric metrics for the post-EVAR configuration are shown in Fig. 8. The values obtained from the numerical simulations are all within the range of in vivo measurements for the post-EVAR configurations [24]. Additionally, it should be highlighted that the variation in the branching angle from inspiration to expiration is higher in the pre-EVAR configuration than in the post-EVAR configuration.

### Morphological Changes in Renal Arteries Induced by Breathing for Different SG Lengths

By measuring the changes in branching angle, it can be observed that the insertion of the stent-graft partially constrains the mobility of the renal arteries during breathing, resulting in a mild reduction in their bending, compared to the pre-EVAR configurations. This impact of the stent-graft on the bending behavior of renal arteries is even more pronounced when the SG length increases, whereas it is reduced by inserting a shorter commercially available stent (Table 5). By analyzing the second metric, the effect is the opposite: an increase in SG length corresponds to an increase in end-stent angle changes, indicating that the end-stent angle augments particularly on expiration, when the natural curvature of the renal artery is reduced due to the presence of a longer stent-graft, leading to greater straightening than for the nominal length (Table 5).

**Table 5:** Quantification of changes in branching angle and end-stent angle from inspiration to expiration (absolute value) for pre-EVAR and post-EVAR geometries, varying SG lengths

		SG length × diameter	Branching angle variation ($^\circ$)	End-stent angle variation ($^\circ$)
		pre-EVAR	9.16	–
Patient 1	RRA	SG 18 × 7 mm	5.45	1.43
		**SG 23 × 7 mm**	5.23	5.98
		SG 27 × 7 mm	2.45	27.12
		pre-EVAR	8.53	–
Patient 1	LRA	SG 18 × 5 mm	5.99	3.25
		**SG 22 × 5 mm**	5.79	10.08
		SG 28 × 5 mm	5.04	13.09
		pre-EVAR	8.59	–
Patient 2	RRA	SG 18 × 6 mm	7.82	8.94
		**SG 22 × 6 mm**	7.49	15.70
		SG 28 × 6 mm	7.13	21.47
		pre-EVAR	11.01	–
Patient 2	LRA	SG 18 × 5 mm	8.30	8.58
		**SG 22 × 5 mm**	7.36	12.50
		SG 28 × 5 mm	5.88	20.73

Nominal SG lengths are highlighted in bold

## Discussion

In the present study, we simulated for the very first time the bending behavior of commercial bridging stent-grafts, defining the material properties for both stent and polymer, necessary to replicate in silico peripheral SG deployment. We built a workflow to perform renal artery stenting, after placing the SG main body in the aorta, leading to the first digital twin for fEVAR including renal stenting and breathing motion. Concerning respiratory motion, the efficacy of the simulations in predicting morphological changes over a respiratory cycle is demonstrated by the agreement with data from in vivo measurements published in the literature [23, 24], for branching angle, end-stent angle, and maximum curvature. For this last metric, the variability is not as wide compared to the data in the literature, probably due to a smaller cohort of patients, since this metric is very sensitive to the actual geometry of patients’ renal arteries. For renal stenting, outcomes of the simulations have been validated via the comparison with post-operative CT scans, since the average difference between the centrelines points of simulations and post-EVAR imaging is consistently below the threshold for all cases examined. Especially, the SG Placement Accuracy Score results to be quite high, above 70% in 3 out of 4 cases. However, differences in simulation outcomes can be observed between the various arteries. With respect to the right renal artery, the curvature of the stent-graft was found to be more pronounced. This discrepancy is likely due to the presence of the inferior vena cava in that anatomical position, which is not considered in the numerical model (Fig. 7). On the other hand, for the left renal artery, and for patient 1 especially, the final placement of the bridging stent-graft is the least accurate, with a SG Placement Accuracy Score of 45%. This weakness is mainly explained by misalignment between the fenestration of the SG main body in the abdominal aorta and the ostium of the left renal artery (Fig. 7a). Due to its curved geometry, a more pronounced morphing deformation was necessary to allow the positioning of the crimped bridging stent-graft and its balloon. In addition, this left renal artery has a much smaller diameter compared to that of the fenestration, which made the additional expansion of the bridging stent-graft protrusion more challenging. For patient 2, the two renal arteries are closer in anatomy, resulting in the insertion of two bridging stent-grafts with the same nominal length (22 mm), and nominal diameter differing by only 1 mm. This results in comparable SG Placement Accuracy Scores, as well as a good initial alignment with the SG main body fenestration. Furthermore, particularly for the left renal artery, its original anatomical conformation is close to straight, which makes the deployment of the bridging stent-graft easier (Fig. 7b). Nevertheless, our numerical simulations can be improved in the future, in order to obtain a higher SG Placement Accuracy Score, reducing the errors due to assumptions of our model, and using specific material properties for the renal arteries as a potential improvement.

Additionally, the balloon was modeled as a cylindrical shell to which pressure is applied, without accounting for the complex design of the actual balloon. This assumption does not allow to simulate the surgical procedure fully. However, as shown by Gervaso et al. [31], it does not significantly affect the stresses on the vascular walls and has the main advantage of reducing the computational cost by simplifying contact interactions, leading to a good compromise between efficiency of the simulations and their reliability.

In addition, by comparing the renal artery configurations between pre- and post-op configurations, we detected a reduction in branching angle variations during breathing (Fig. 8a). This highlights the impact of the stent-grafts which limits the physiological bending of the renal arteries during breathing due to its stiffness compared to the vessel walls. These findings align with the observations by Tran et al. [24] who as first quantified the consequences of fEVAR bridging stent placement on branching angulation, linking this phenomenon to the stiffness provided by the SG main body. Our results are also consistent with the findings of Cheng et al. who noticed a reduction in branching angle deformation during breathing in post-op configurations [25, 38]. This variation is even significant if we vary the length of the stent-graft to test other potential choices for surgeons in the pre-operative pre-planning phase. It is worth noting that we only used actual lengths of commercially available SGs, as the goal is to assist surgeons in the decision-making process, by predicting potential complications. An increase in stent-graft length leads to an even more significant reduction in branching angle and an increase in end-stent angle, altering the morphology of the arteries (Table 5). A higher end-stent angle variation during breathing was observed as the length of the bridging stent-graft increases. This variation is the consequence of the partial straightening that the renal arteries undergo with stenting (Table 5). The impact of the stiff stent-graft on the morphology is even more evident during expiration, when the bending of the artery occurs.

To the authors knowledge, this is the first study to describe the impact of different bridging SG lengths on renal morphology and on breathing motion, acting as a potential tool for surgical pre-planning. This simulation-based approach can effectively support clinical decision-making, particularly in selecting the appropriate stent-graft length, minimizing the risk of complications such as occlusions, kinking, or endoleaks associated with an inappropriate SG length. Accurately predicting renal artery movement and renal stent interaction with digital twin technology will help clinicians better select the appropriate bridging stent to reduce the aforementioned complications. In the context of modeling renal arteries, we assume their mechanical properties to be identical to that of the aorta (with a different thickness). This simplification of equivalent mechanical properties may partially impact the accuracy of the simulations but is made in the absence of specific data on bending mechanical behavior of human renal arteries in the literature. Experimental testing of renal artery mechanical properties presents significant challenges, due to the difficulty in obtaining in vivo tissue samples. We acknowledge that renal arteries differ from the aorta, particularly in terms of bending mechanical behavior, as the aorta does not experience the same deflection phenomena associated with breathing. Furthermore, another limitation of our study is that, as we do not have 4DCT (time resolved) scans, clinical images are acquired when patients are not in a free-breathing condition, but in a breath-hold condition, according to the instructions of the radiologists who recorded the images in inspiration and exhalation.

This study developed the first digital twin of fEVAR procedures that incorporates renal stenting through the deployment of bridging stent-grafts of different lengths. To achieve this, we overcome several challenges, including the identification of the mechanical properties of peripheral stent-grafts and the accurate simulation of bridging stent-graft insertion into the renal arteries, replicating the unique geometry of the flared stent-grafts. The insertion of peripheral stent-grafts of varying lengths was evaluated, accounting for respiratory motion, and demonstrating that increased stent-graft length reduces the variation in branching angle due to the stiffness of the stent. Future research will aim to expand the patient cohort and adapt the digital twin to the geometric variability of the patient population. The model will further be tested and validated with various anatomies, especially from cases with renal stent complications during follow-up. CFD simulations will be conducted to investigate the hemodynamics in the renal arteries, focusing on the changes due to breathing and the insertion of the bridging stent-grafts. This hemodynamics study will provide insights about possible complications.

## Appendix

The boundary conditions applied to simulate the respiratory motion derived from patient-specific CT scans are summarized in Table 6. These include the displacement components along the cranio-caudal (CC), antero-posterior (AP), and right–left (RL) directions at the distal ends of the renal arteries, from inspiration to expiration.

**Table 6:** Cranio-Caudal (CC), Antero-Posterior (AP), Right–Left (RL) renal artery boundary conditions to simulate expiration from inspiration

Patients	Left renal artery (mm)			Right renal artery (mm)		
	CC	AP	RL	CC	AP	RL
Patient 1	$-$15.8	$-$8.0	$-$1.1	$-$7.4	$-$3.9	0.5
Patient 2	$-$16.7	$-$9.9	$-$0.1	$-$17.7	$-$3.9	1.8

To test the influence of different renal artery material properties on simulation outcomes, a sensitivity analysis was performed by varying the mechanical properties by ±20%. Table 7 reports the changes of renal branching angle in response to such variations.

**Table 7:** Renal material properties sensitivity analysis

			Branching angle variation ($^\circ$)	% Variation
Patient 1		Pre-EVAR	9.16	–
	RRA	Pre-EVAR (Mat. Prop. $-$20%)	9.19	+ 0.3%
		Pre-EVAR (Mat. Prop. + 20%)	8.26	$-$9.8%
Patient 1		Pre-EVAR	8.53	–
	LRA	Pre-EVAR (Mat. Prop. $-$20%)	9.04	+ 5.6%
		Pre-EVAR (Mat. Prop. + 20%)	8.23	$-$3.6%

To verify the impact of different breathing boundary conditions on branching angle variations, a sensitivity analysis was performed by applying variations of ±1.5 mm, in agreement with the 3-mm potential inaccuracies in CT scan reconstructions, defined in the “Results” section (Table 8).

**Table 8:** Breathing boundary conditions sensitivity analysis

			Branching angle variation ($^\circ$)	% Variation
Patient 1		Pre-EVAR	9.16	–
	RRA	Pre-EVAR (BC $-$1.5 mm)	7.59	$-$17.1%
		Pre-EVAR (BC + 1.5 mm)	9.60	+ 4.8%
Patient 1		Pre-EVAR	8.53	–
	LRA	Pre-EVAR (BC $-$1.5 mm)	7.60	$-$10.9%
		Pre-EVAR (BC + 1.5 mm)	9.23	+ 8.2%
